# Fibrodysplasia Ossificans Progressiva: Clinical and Genetic Aspects

**DOI:** 10.1186/1750-1172-6-80

**Published:** 2011-12-01

**Authors:** Robert J Pignolo, Eileen M Shore, Frederick S Kaplan

**Affiliations:** 1 Department of Medicine, Perelman School of Medicine, University of Pennsylvania, Philadelphia, Pennsylvania 19104 USA; 2 Department of Orthopaedic Surgery, Perelman School of Medicine, University of Pennsylvania, Philadelphia, Pennsylvania 19104 USA; 3 Department of Genetics, Perelman School of Medicine, University of Pennsylvania, Philadelphia, Pennsylvania 19104 USA

**Keywords:** fibrodysplasia ossificans progressiva, myositis ossificans, heterotopic ossification, progressive osseous heteroplasia, trauma, toe malformation, Activin A receptor type I/Activin-like kinase 2 (ACVR1/ALK2)

## Abstract

Fibrodysplasia ossificans progressiva (FOP) is a severely disabling heritable disorder of connective tissue characterized by congenital malformations of the great toes and progressive heterotopic ossification that forms qualitatively normal bone in characteristic extraskeletal sites. The worldwide prevalence is approximately 1/2,000,000. There is no ethnic, racial, gender, or geographic predilection to FOP. Children who have FOP appear normal at birth except for congenital malformations of the great toes. During the first decade of life, sporadic episodes of painful soft tissue swellings (flare-ups) occur which are often precipitated by soft tissue injury, intramuscular injections, viral infection, muscular stretching, falls or fatigue. These flare-ups transform skeletal muscles, tendons, ligaments, fascia, and aponeuroses into heterotopic bone, rendering movement impossible. Patients with atypical forms of FOP have been described. They either present with the classic features of FOP plus one or more atypical features [FOP plus], or present with major variations in one or both of the two classic defining features of FOP [FOP variants]. Classic FOP is caused by a recurrent activating mutation (617G>A; R206H) in the gene *ACVR1/ALK2 *encoding Activin A receptor type I/Activin-like kinase 2, a bone morphogenetic protein (BMP) type I receptor. Atypical FOP patients also have heterozygous *ACVR1 *missense mutations in conserved amino acids. The diagnosis of FOP is made by clinical evaluation. Confirmatory genetic testing is available. Differential diagnosis includes progressive osseous heteroplasia, osteosarcoma, lymphedema, soft tissue sarcoma, desmoid tumors, aggressive juvenile fibromatosis, and non-hereditary (acquired) heterotopic ossification. Although most cases of FOP are sporadic (noninherited mutations), a small number of inherited FOP cases show germline transmission in an autosomal dominant pattern. At present, there is no definitive treatment, but a brief 4-day course of high-dose corticosteroids, started within the first 24 hours of a flare-up, may help reduce the intense inflammation and tissue edema seen in the early stages of the disease. Preventative management is based on prophylactic measures against falls, respiratory decline, and viral infections. The median lifespan is approximately 40 years of age. Most patients are wheelchair-bound by the end of the second decade of life and commonly die of complications of thoracic insufficiency syndrome.

## Disease name and synonyms

Fibrodysplasia ossificans progressiva (ORPHA337)

Myositis ossificans progressiva (ORPHA337)

### Definition

**Figure 1:**
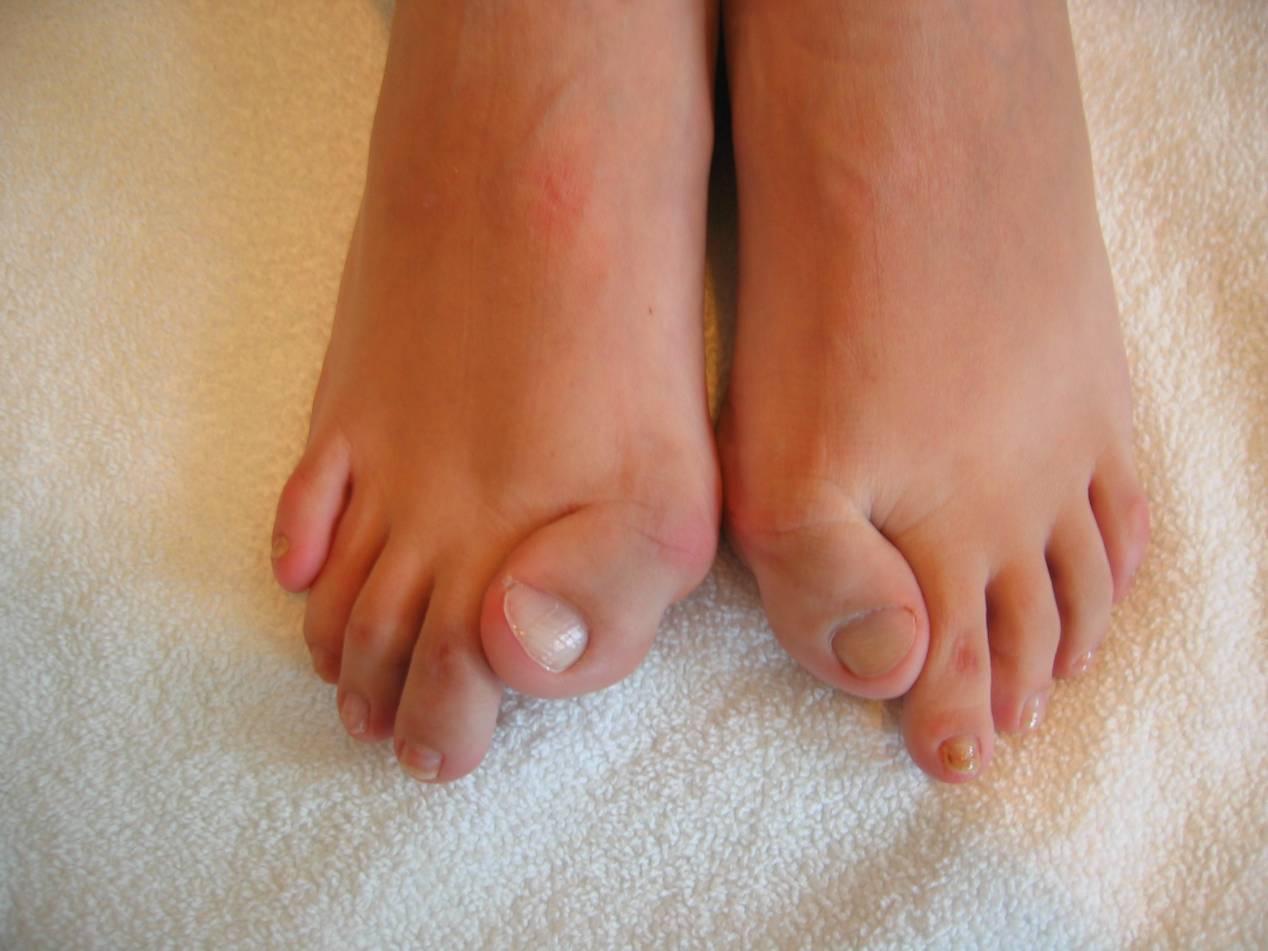
**Characteristic malformed great toes and hallux valgus**.

**Figure 2:**
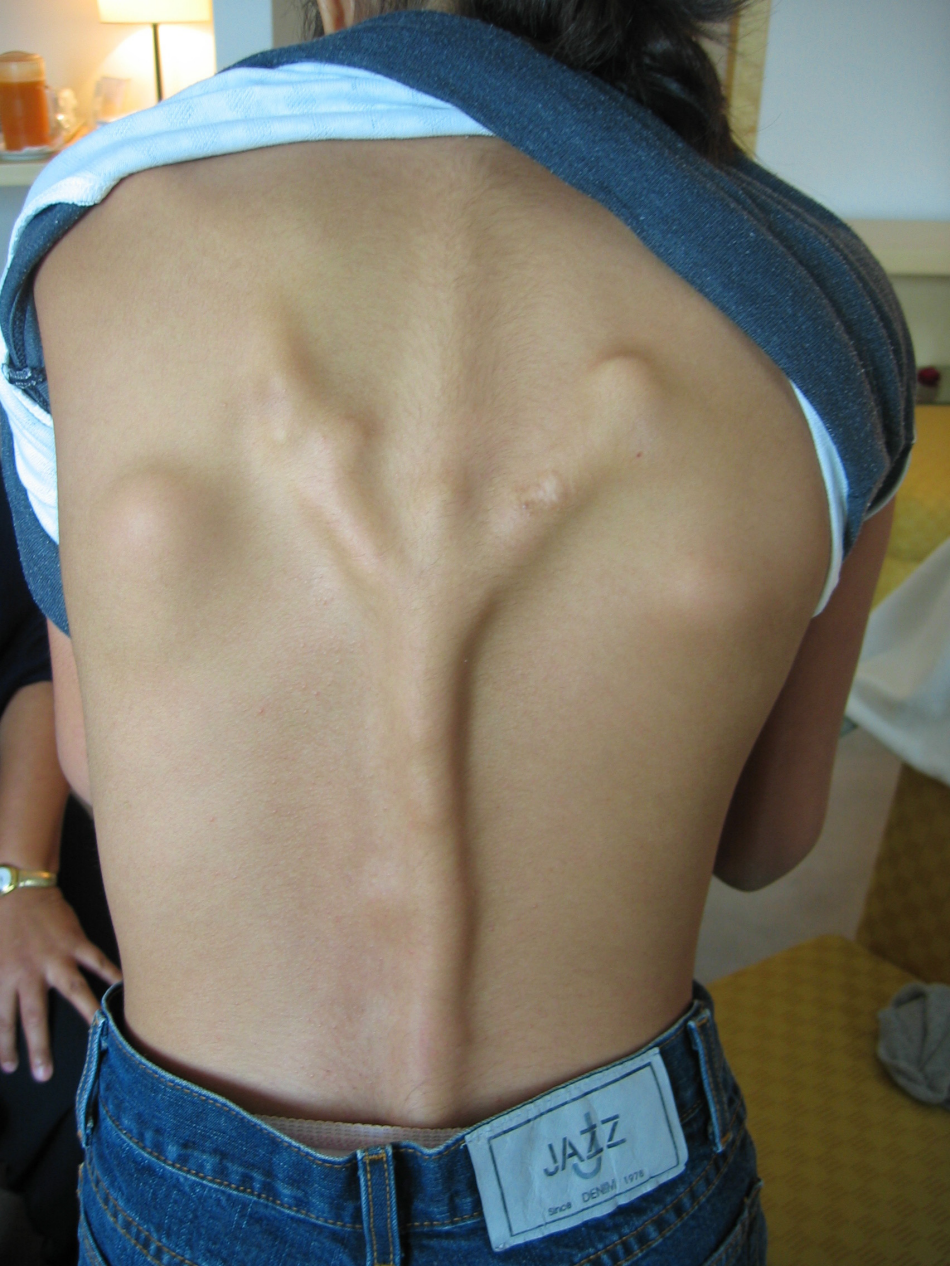
**Extensive heterotopic ossification on the back of an FOP patient**.

Fibrodysplasia ossificans progressiva (FOP) (Mendelian Inheritance in Man [MIM] #135100) [1] is a severely disabling heritable disorder of connective tissue characterized by congenital malformations of the great toes (hallux valgus, malformed first metatarsal, and/or monophalangism) (Figure 1) and progressive heterotopic ossification (HO) that forms qualitatively normal bone in characteristic extraskeletal sites (Figure 2). Involvement of dorsal, axial, cranial, and proximal anatomic locations precede involvement of ventral, appendicular, caudal, and distal areas of the body.

### Epidemiology

FOP is very rare with a worldwide prevalence of approximately 1 case in 2 million individuals. No ethnic, racial, or geographic predisposition has been described [2].

### Clinical Description

**Figure 3:**
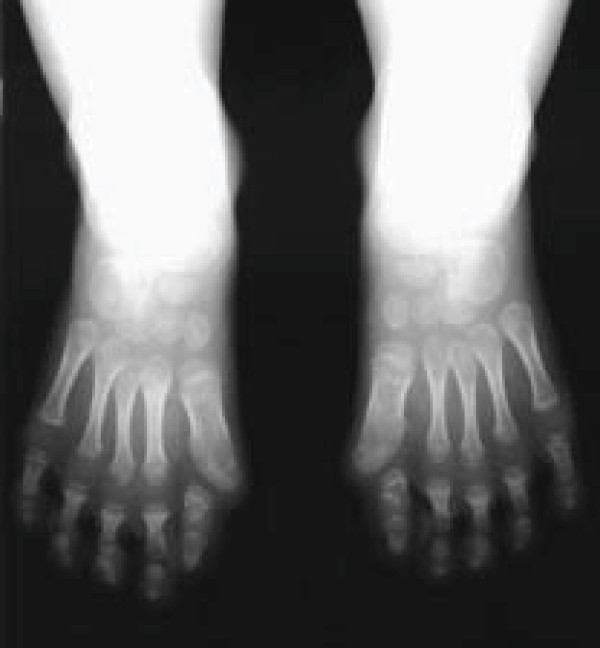
**Anteroposterior radiograph of the feet of a three-year old child showing symmetrical first toe malformations. ** Reprinted from reference 14. Authors retain copyright.

FOP is the most disabling condition of ectopic skeletogenesis. Children who have FOP appear normal at birth except for congenital malformations of the great toes (Figures 1 and 3).

**Figure 4:**
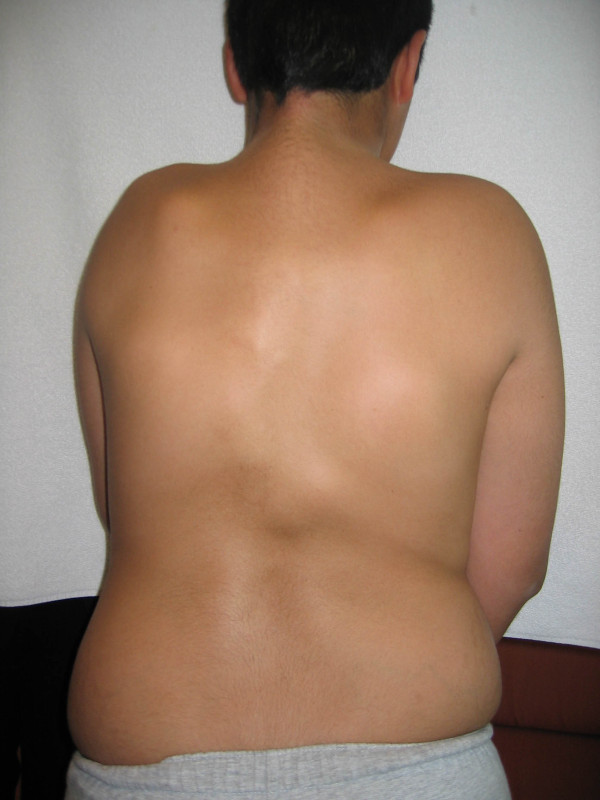
**Tumor-like swellings on the back representing early FOP flare-ups**.

**Figure 5:**
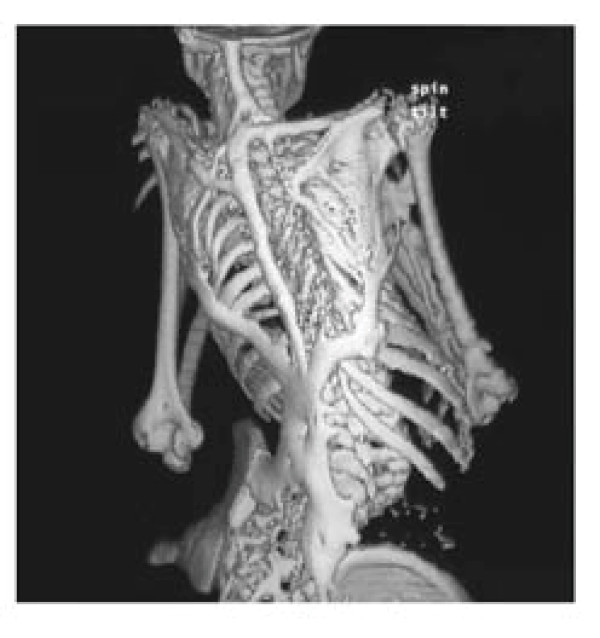
**Three-dimensional reconstructed computed tomography (CT) scan of the back of a twelve-year old child showing extensive heterotopic ossification typical of FOP**. Reprinted from reference 14. Authors retain copyright.

Typically, during the first decade of life, sporadic episodes of painful soft tissue swellings (flare-ups) occur [3] and are commonly mistaken for tumors (Figure 4) [4]. Although some exacerbations spontaneously regress, flare-ups can transform skeletal muscles, tendons, ligaments, fascia, and aponeuroses through an endochondral process into ribbons, sheets, and plates of heterotopic bone (Figures 2 and 5) that span the joints, lock them in place, and render movement impossible [5,6]. Progressive episodes of HO occur in specific anatomic patterns, and are typically seen first in the dorsal, axial, cranial, and proximal regions of the body and later in the ventral, appendicular, caudal, and distal regions [7]. Attempts to remove this heterotopic bone usually lead to episodes of explosive new bone formation. Trauma such as minor soft tissue injury, muscular stretching, over-exertion and fatigue, intramuscular immunizations, injections for dental work, falls, and influenza-like illnesses can induce flare-ups of the condition. Immobility is cumulative and most patients are wheelchair-bound by the end of the second decade of life.

The diaphragm, tongue, and extra-ocular muscles are spared from HO. Cardiac muscle and smooth muscle are also spared in FOP. Neck stiffness is an early finding and can precede the appearance of HO at that site. Cervical spine abnormalities include large posterior elements, tall narrow vertebral bodies, and fusion of the facet joints between C2 and C7 [8]. The cervical spine often becomes ankylosed early in life. Other skeletal features associated with FOP are short malformed thumbs, clinodactyly, short broad femoral necks, and proximal medial tibial osteochondromas [9-11].

In addition to progressive immobility, other life-threatening complications include severe weight loss following ankylosis of the jaw, as well as pneumonia and right-sided heart failure resulting from thoracic insufficiency syndrome (TIS) [12]. Features of FOP that contribute to TIS include costovertebral malformations with orthotopic ankylosis of the costovertebral joints, ossification of intercostal muscles, paravertebral muscles and aponeuroses, as well as progressive spinal deformity including kyphoscoliosis or thoracic lordosis.

Limb swelling seen with flare-ups may be substantial and lead to extra-vascular compression of nerves and tissue lymphatics. Some patients with advanced FOP involving the lower limbs have venous stasis and/or lymphedema. In both scenarios, studies to exclude deep vein thrombosis may be difficult to obtain and interpret. Limb swelling is often difficult to treat.

Hearing loss is usually conductive and may be due to middle ear ossification, but in some patients the hearing impairment is sensorineural, involving the inner ear, cochlea, or the auditory nerve [13].

Among patients with FOP-like HO and/or toe malformations, a small number of patients has clinical features unusual for FOP [11]. These atypical FOP patients are categorized as FOP-plus (classic defining features of FOP plus one or more atypical features) and FOP variants (major variations in one or both of the two classic defining features of FOP, such as normal great toes or severe reduction deficits of digits). Atypical features associated with FOP-plus include intraarticular synovial osteochondromatosis of hips, degenerative joint disease of hips, sparse/thin scalp hair (more prominent in second decade), mild cognitive impairment, severe growth retardation, cataracts, retinal detachment, childhood glaucoma, craniopharygioma, persistence of primary teeth in adulthood, anatomic abnormalities of the cerebellum, diffuse cerebral dysfunction with seizures, polyostotic fibrous dysplasia, primary amenorrhea, aplastic anemia, hypospadias, and cerebral cavernous malformations [11].

### Aetiology

A large body of work has supported dysregulated bone morphogenetic protein (BMP) signaling in the pathogenesis of FOP. A single common heterozygous mutation (617G>A; R206H) has been identified in the cytoplasmic domain of activin receptor IA/activin-like kinase 2 (ACVR1/ALK2), a BMP type I receptor, in affected individuals of five small multigenerational families and in all sporadically affected individuals with the features of classic FOP [14]. All atypical FOP patients have heterozygous *ACVR1 *missense mutations in conserved amino acids [11]. Novel *ACVR1 *mutations have been described for FOP variants and in two cases of FOP plus [11]. To date, all *ACVR1 *mutations evaluated for enhanced BMP signaling are gain-of-function mutations [11,15].

Mounting evidence suggests that involvement of the inflammatory component of the immune system plays a critical role in FOP [16]. The presence of macrophages, lymphocytes and mast cells in early FOP lesions, macrophage and lymphocyte-associated death of skeletal muscle, flare-ups following viral infections, the intermittent timing of flare-ups, and the beneficial response of early flare-ups to corticosteroids support involvement of the innate immune system in the pathogenesis of FOP [17].

### Diagnosis and diagnostic methods

Clinical suspicion of FOP early in life on the basis of malformed great toes can lead to early clinical diagnosis and the avoidance of harmful diagnostic and treatment procedures. Routine biochemical evaluations do not contribute to making the diagnosis. Plain x-rays can substantiate more subtle great toe abnormalities and the presence of HO. Abnormal findings on bone scan occur before HO can be detected by conventional radiography, but sophisticated imaging studies are generally superfluous from a diagnostic standpoint. Confirmatory genetic testing is available on a clinical and research basis at several laboratories [18].

### Differential Diagnosis

FOP must be distinguished from other genetic conditions of heterotopic ossification (HO), and nonhereditary (acquired) HO. Progressive osseous heteroplasia (POH) is a rare genetic condition of progressive HO defined clinically by cutaneous ossification that usually presents during childhood and progresses to involve subcutaneous and deep connective tissues, including muscle and fascia, in the absence of multiple features of Albright hereditary osteodystrophy (AHO) or hormone resistance [19]. FOP is differentiated from POH by congenital malformation of the great toes, preosseous inflammation or "flare-ups" and the lack of cutaneous ossification.

Acquired HO commonly follows severe trauma, and can be observed at any age but is rare in young children [20]. Acquired HO tends to occur at periarticular sites or at sites of blunt trauma or localized injury.

FOP is commonly misdiagnosed as aggressive juvenile fibromatosis, lymphedema, or soft tissue sarcoma [4]. Other diagnostic considerations are lymphoma, desmoids tumors, isolated congenital malformations, brachydactyly (isolated), and juvenile bunions.

### Genetic counseling

Most cases of FOP arise as a result of a spontaneous new mutation. Genetic transmission is autosomal dominant and can be inherited from either parent. Within a family, inherited FOP can show variable expression. If a parent has FOP, the chance that a child will inherit FOP is 50%.

There are substantial life-threatening risks to both the mother and child that women with FOP might encounter during pregnancy. Specific risks to the mother include, but are not limited to: (1) risk of FOP flare-ups during pregnancy; (2) risk of breathing difficulties during the latter part of pregnancy; (3) risk of childbirth complications not uncommon in mothers without FOP. [For example, Caesarian section is necessary due to the pelvic deformity, joint fusions, and decreased plasticity of the birth canal that will not safely accommodate a normal vaginal delivery]; (4) risk of the general anesthesia for Caesarian delivery; and (5) risk of phlebitis and pulmonary embolism. The authors are unaware if the use of amniocentesis has caused complications in the mother. Specific risks to the child include, but are not limited to: (1) risk that the child may have FOP; (2) risk of prematurity; (3) risk of severe fetal distress; (4) risk of cerebral palsy; (5) risk of complications from general anesthesia. At delivery, there should be a team skilled in resuscitation of high risk infants.

### Antenatal diagnosis

Prenatal testing is not routinely available for FOP.

### Management including treatment

Flare-ups of FOP are sporadic and unpredictable, with great individual variability of disease progression.

### Preventative measures

In children, restriction of activity to less physically interactive play may be helpful to reduce falls, but strict avoidance of high-risk circumstances is not only impractical but may also prevent patients from optimizing their level of function. Restriction of activity also compromises independence and may be unacceptable to patients. Physical rehabilitation should be focused on enhancing activities of daily living through approaches that avoid passive range of motion which could lead to disease flare-ups.

Acceptable modification of activity, improvement in household safety, use of ambulatory devices, and use of protective headgear are all strategies to prevent falls and minimize injury when falls occur. Prophylactic measures that minimize respiratory decline (e.g., incentive spirometry) and prevent influenza and pneumonia (i.e., appropriate immunizations) may decrease morbidity and mortality associated with thoracic insufficiency syndrome.

Intramuscular injections, including immunizations, must be avoided, but vaccinations administered by subcutaneous injection and routine venipuncture pose little risk. Great care is necessary in administering dental care, particularly in avoiding overstretching of the jaw and intramuscular injections of local anesthetic [21]. Preventive oral and dental health care measures are essential. Successful anesthesia in mandibular primary teeth can be achieved by infiltration through the dental pulp. Interligamentary infiltration may be helpful, if performed carefully. General anesthesia with awake nasotracheal fiberoptic intubation may be needed for dental care in some patients.

Conductive hearing loss is common and children should generally have audiology evaluations at least every other year. Hearing aids can diminish developmental problems in children.

Surgical attempts to remove heterotopic bone will provoke explosive new bone growth and should not be attempted.

### Pharmacotherapy

Corticosteroids are indicated as first-line treatment at beginning of flare-ups. A brief 4 day course of high-dose corticosteroids, started within the first 24 hours of a flare-up, may help reduce the intense inflammation and tissue edema seen in the early stages of the disease. The use of corticosteroids should be restricted to treatment of flare-ups that affect major joints, the jaw, or the submandibular area. Corticosteroids should not generally be used for the symptomatic treatment of flare-ups that involve the back, neck, or trunk due to the long duration and recurring nature of these flare-ups and the difficulty in assessing the true onset of such flare-ups. A typical dose of prednisone is 2 mg/kg/d, administered as a single daily dose. If a second course of corticosteroids becomes necessary, this should be followed by a slow taper.

When prednisone is discontinued, a nonsteroidal anti-inflammatory drug (NSAID) or cox-2 inhibitor (in conjunction with a leukotriene inhibitor) may be used symptomatically for the duration of the flare-up. The use of mast cell inhibitors and aminobisphosphonates can be used at the physician's discretion.

Many flare-ups are extremely painful and may require a brief course of well-monitored narcotic analgesia in addition to the use of NSAIDs, cox-2 inhibitors, and oral or intravenous glucocorticoids. The cautious short-term use of muscle relaxants such as cyclobenzaprine (Flexeril), metaxalone (Skelaxin), or Lioresal (Baclofen) may help to decrease muscle spasm and maintain more functional activity.

For a complete description of medications used in the treatment of FOP, including dosing and potential major side effects, refer to the current treatment guidelines at the International Fibrodysplasia Ossificans Progressiva Association website [22].

### Prognosis

The median lifespan is approximately 40 years of age [23]. Most patients are wheelchair-bound by the end of the second decade of life and commonly die of complications of thoracic insufficiency syndrome [23].

### Unresolved questions

The discovery of the specific FOP gene mutation and the results of preliminary studies over the past several years show clearly that while the specific *ACVR1/ALK2 *gene mutation may be necessary to cause heterotopic ossification in FOP, it is not sufficient for inducing flare-ups of the disease that lead to progressive and cumulative disability. The required role of the immune system, the responses of resident progenitor cells, and the biochemical aberrations of the soft tissue microenvironment all appear to be intimately involved in the induction of flare-ups and episodic progression of the disease. The investigation of relevant cells and microenvironmental factors is necessary to elucidate the complex pathophysiology of FOP.

## Consent

Written informed consent could not be obtained for the cases illustrated in Figures 1, 2, and 4, since the patients are untraceable from the photographs taken. We believe this review contains important clinical examples, which could not be as effectively made in any other way. We expect that reasonable persons would not object to the publication of these photographs since details of each patient remain anonymous.

## Competing interests

The authors declare that they have no competing interests.

## Authors' contributions

RJP conceived, designed and drafted the manuscript. EMS and FSK contributed to the critical review and revision of the manuscript. All authors read and approved the final manuscript.
